# Effect of cognitive behavioral therapy program on mental health status among medical student in Palestine during COVID pandemic

**DOI:** 10.1186/s12888-022-03915-1

**Published:** 2022-05-02

**Authors:** Ahmad Hanani, Manal Badrasawi, Souzan Zidan, Marah Hunjul

**Affiliations:** 1 Department of Public Health, Faculty of Medicine and Health Sciences, An-Najah National University, PO. Box 7, Nablus, West Bank Palestine; 2 Department of Nutrition and Food Technology, Faculty of Agriculture and Veterinary Medicine, An-Najah National University, PO. Box 7, Tulkarm, West Bank Palestine; 3 Department of Nutrition and Food Technology, Faculty of Agriculture, Hebron University, PO. Box 40, Hebron, West Bank Palestine; 4 Medicine Program, Faculty of Medicine and Health Sciences, An-Najah National University, PO. Box 7, Nablus, West Bank Palestine

**Keywords:** Mental illness, Medical students, General Health Questionnaire-12, Cognitive behavior therapy

## Abstract

**Background:**

The COVID-19 pandemic had a profound psychological influence on everyone in society, and the impact it had on students, particularly medical students, cannot be underestimated. The main purpose of this study is to (1) determine the prevalence of mental disorders among medical students and their associated factors, and (2) examine the effectiveness of cognitive behavior therapy on mental health problems among medical students.

**Methods:**

Between March and May 2021, we conducted a randomized controlled study on two phases among medical students at An-Najah National University. Data were collected using an online questionnaire and the Arabic version of the 12-item General Health Questionnaire (GHQ-12). We also used the MEDAS tool to assess their Mediterranean Diet (MD) adherence. In the second phase, sixty-six students were recruited and assigned randomly to control and intervention groups. Intervention impact was assessed using 12-item General Health Questionnaire at two-time points; baseline, and 8 weeks post-intervention. The interventional model used was cognitive behavioral therapy, and the control group received no treatment.

**Results:**

A total of 329 students were included in the analysis of the first phase of the study. Approximately 28% of students had mental health problems. We found a significant relationship between good mental health status with a higher level of physical activity level, longer sleeping hours, and shorter entertainment time (*p* < 0.05). In the second phase of the study, a total of 91 students were included. Overall, using the CBT program showed a significant improvement in the outcome measures. At 8 weeks post-intervention, students had lower scores on total GHQ-12, depression, anxiety, and social dysfunction.

**Conclusion:**

These findings propose that adequate attention must be paid to the mental health of medical students and that CBT programs can be used for the management of mental health problems among medical students.

**Supplementary Information:**

The online version contains supplementary material available at 10.1186/s12888-022-03915-1.

## Introduction

The first case of coronavirus disease 2019 (COVID-19) stood out from Wuhan, China in December 2019 [1]. In Palestine, the first confirmed case occurred on March 5, 2020, after a group of Greek tourists who visited a hotel in late February tested positive for SARS-CoV-2. According to the health minister of Palestine, by May 2021, there have been 333,810 and 3,720 deaths recorded in Palestine [2].

The highly contagious nature of this virus needed instant lockdowns and quarantines. Globally, these preventive actions had a great impact on students of all ages [3]. To help contain the spread of COVID-19, all schools and universities in Palestine transitioned classes to remote instruction, following the declaration of a state of national emergency by the Palestinian National Authority on 5th March 2020.

As might be expected, not all students and lecturers were ready for this fast transition, and many lacked enough access to suitable resources and infrastructure. This gave rise to unforeseen and new handicaps for numerous students, and in some cases brought about a dramatic perturbation of the educational process [4, 5]. The majority of students were obliged to remain at their houses and to study the required subjects by themselves, and many of them had restricted access to study resources, and the minority of them had no chance for interpersonal interactions with either their lecturers or their classmates. This exceptional condition resulted in qualms for the future, depression, anxiety, and isolation. Recent research noticed that this whole condition resulted in considerable stress among undergraduates [4]. Further studies disclosed growing reports of depression, anxiety, and loneliness as a result of lockdown enjoins required to prevent the spread of the coronavirus [6]. These forms of stress have an undesirable influence and can also give rise to poor mental health [7].

The COVID-19 outbreak had a significant psychological impact on all members of society, and the influence it had on students, particularly medical students, cannot be underestimated [8]. These students’ academic, recreational, personal, and social lives were all disrupted as a result of their regular exposure to COVID-19 patients. Such disruptions among the students caused fear, anxiety, frustration, boredom, and a feeling of isolation from the rest of the world [9].

In the current study, we concentrated specifically on medical students. Medical school is a quite demanding stage in a medical student’s life where a lot is foreseeing from students. Besides coping with naturalistic stressors in life, medical students have to cope with the shortage of free time, the intricacy of medical science, seeking job opportunities at the end of studying years, and the financial debt that they have to incur [10]. During the first year of medical school, medical students have equivalent mental morbidity in comparison to the general population and non-medical students [11, 12]. However, as medical students progress in their studies, they will become exposed to increased levels of psychological distress mental morbidity compared to non-medical students and the general population [13].

Globally, roughly one-third of the medical students have either depressive symptoms or depression [14, 15]. Alongside depression, psychosomatic disorder and anxiety frame a prominent mental health problem [16]. Furthermore, medical students are more prone to developing eating disorders as well [17].

Mental morbidity among medical students has been recognized in several countries including Egypt [18], Saudi Arabia [19], Iran [20], Pakistan [10, 21], China [22], Malaysia [23], India [24–26], Poor mental health also notices to be correlated with suicidal ideation and fatigue [27], substance abuse [10], and earnest thoughts of leaving medical school [28].

Numerous studies showed that women medical students experience more stress than men medical students during their studies [29], whereas other studies indicated no relationship between race and psychological distress among medical students [30, 31]. Marital status has also been found to be related to medical students’ mental health, married students being less probable to suffer from psychological distress [32]. Besides that, low academic performance has also been related to psychological distress [33, 34].

Cognitive Behavior Therapy is evidence-based psychotherapy, and it had been used globally in the prevention and treatment of psychological as well as physical issues [35, 36]. Cognitive-behavioral therapy includes methods that aim to help a person to identify his stress levels and modify his beliefs and behaviors and such methods include cognitive restructuring, behavioral changes, and social support [37]. It helps a person to eliminate or reduce psychological distress symptoms and helps the individual to return to normal day-to-day life. Many studies had found that after receiving cognitive behavior therapy, there is a significant drop in anxiety, improvement in somatic symptoms and psychological stress, and an increase in quality of life [38]. Studies have shown that Cognitive behavior therapy is the most economical and effective psychotherapy in reducing and relieving psychological distress [39, 40].

Presently, there are no programs in the medical school curriculum of Palestine to screen for poor mental health. Comprehending medical students’ mental health will foster the evolvement and incorporation of student health programs and specific educational interventions to avoid unfavorable consequences of poor mental health. Therefore, the main aim of the current randomized controlled trial was to evaluate the effectiveness of cognitive behavior programs for the treatment of psychological problems during the recent stay-at-home quarantine among medical students at one private medical school in Palestine who were categorized with mental health issues during their participation at the screening phase of the current study, and they did not ask for help or treatment before, because they were shy, and fear from the stigma.

### Methodology

Our study was done in two phases; In the first phase, we determined the prevalence of mental health problems and their associated factors among a representative sample of medical students at An-Najah National University, Palestine, using a 12-item General Health Questionnaire (GHQ-12). In the second phase, we explored the overall effect of a specifically designed cognitive behavior therapy program on mental health among medical students who were categorized with mental health issues from the screening phase. Ethical approval was obtained from the Institutional Review Board at An-Najah National University before the initiation of this study.

### Data collection

All participants were briefed about the study design and objectives, and they were informed about the type of data that would be collected. Data were collected using a self-administrated online questionnaire. Besides, verbal and signed consent was obtained from all participants prior to the initiation of the study. Data were collected from March to May 2021. The inclusion criteria for the first phase of the study were participants aged 18 years and over and willing to participate and to provide all the required data. Participants who had a tendency towards psychological problems (General Health Questionnaire-12 score 15 or above) and were able to attend all CBT program sessions were involved in the second phase of the study. Students who were taking either antidepressant drugs or psychotropic drugs were excluded from the study.

### Sample size calculation

In the screening phase, the participants were recruited by simple random sampling. The representative sample size calculation was based on the following formula [21]:

$$\mathrm{n}={\left(\frac{\mathrm{z}}{\mathrm{E}}\right)}^{2}\mathrm{p}\left(1-\mathrm{p}\right).$$

Using 95% confidence level (z = 1.96), estimation error (E = 0.05), and the prevalence (*p* = 0.5), this formula gives *n* = 320. Considering drop out the study sample was increased to 350 participants. For the intervention phase, the sample size was calculated using the Chan formula for sample size calculation in randomized controlled clinical trials. The mean and standard deviations were taken from a similar randomized clinical trial [41], conducted to determine the improvement in depressive symptoms following cognitive-behavioral therapy. It showed significant improvement among the intervention group. Ninety percent power and 0.05 level of confidence were assumed to calculate the sample size. The final sample size calculation deemed required was 30 students in each group.

### The intervention program

The Cognitive behavioral therapy (CBT) program is considered a psycho-social intervention that aims to improve mental health by focusing on changing cognitive distortions (e.g. thoughts, beliefs, and attitudes) and behaviors. Therefore, we used this program to reduce medical students’ psychological problems during distance learning at the time of COVID-19. The CBT program focused on topics summarized in Table 1. The psychotherapist followed a training manual. Each 60-min weekly session via zoom program (online session) consisted of the following: lecture, discussion, and training. The use of the CBT program has been reviewed and approved by experts in the field of the study.

**Table 1:** Topics of 8-Weeks CBT Intervention for medical students

Title of the session	Number of session	The topic of the session
Introducing program therapy	1	Provide a brief about the program and number of sessions will be, agreement form, date of each session….etc. + Pre-assessment
Psycho-education	2	Explain the stress, anxiety, depression &how it affects our body/self …etc Know when anxiety happens, and what situations create and where
Introducing negative thinking & Thinking Exposure	3 + 4	Explain it, and how it affects us Learn to explain the situation and how to think again in different ways
Negative thinking becomes positive	5 + 6	Learn how to change it to be positive Explain the strategies to face & replace them with a positive thing
Relaxation & Facing the anxiety situation	7	Train him on how to reduce his fear and anxiety during the anxiety situation Learn how to face the anxiety …. etc
End of program & post-assessment	8	Make post-assessment and close the program

### Ethics approval

Ethical approval was obtained from the Institutional Review Board "IRB" at An-Najah National University in Nablus-Palestine and was performed in compliance with the Helsinki Declaration for research humans. The trial has been registered and approved by the TCTR committee on 29/10/2021. The TCTR identification number is TCTR20211029002 (https://www.thaiclinicaltrials.org/show/TCTR20211029002).

Table 1 shows the sessions of the intervention program according to the eight weeks of the CBT intervention program.

### Procedure

Participants who met the inclusion criteria for the second phase of the study and gave informed consent underwent a baseline assessment for their mental health, then they were randomized by computer algorithm into an intervention group (*n* = 34) and a control group (*n* = 32). Participation was free of charge and no reimbursement was offered for participation. Each participant was given a code in order to ensure their attendance at the sessions and to add their assessment at the end of the intervention phase. And to improve clinical outcomes, the psychotherapist was sending WhatsApp messages for participants to confirm the day and time of the session.

Each participant in the control group was only provided with general information about mental health via WhatsApp messages. Furthermore, the psychotherapist was sending SMS messages for participants to check whether they had any inquiries. At the end of the 8-week intervention, students’ mental health status was assessed immediately by GHQ-12.

### Instruments

A pretested, structured questionnaire was used to collect the data from the participants online. The collected data included sociodemographic data (e.g., gender, marital status, academic year, place of residence, living status, working status, and monthly income) and medical history data (e.g., does the participant suffer from chronic disease?, continuous use of drugs, whether the participant underwent a previous surgery, type of surgery, and when?), and data related to lifestyle (e.g., whether the participant smoke, the duration of smoking, duration of sleeping per day, duration of using electronics for studying purposes per day, and duration of using electronics for entertainment purposes per day).

An international physical activity level questionnaire was also used to assess participants’ physical activity levels [42]. Furthermore, participants were asked to record their anthropometric measurements (i.e., height and weight). Body mass index was calculated as (body weight in kilograms divided by height squared in meter (kg/m2), thereafter classified according to WHO cut-off points [43].

Adherence to the Mediterranean diet was measured by a 14- item Mediterranean Diet Adherence Screener (MEDAS), which consists of 14 questions about food consumption frequency and two questions about food intake habits specific to the Mediterranean diet [44]. If the intake condition was not met, 0 points were recorded for the food category. The final score ranged from 0 to 14 [45]. A score of ≤ 5 points indicates weak adherence, a score of 6–9 points indicates moderate adherence, and a score of ≥ 10 points indicates high adherence [19, 44].

For the measurement of mental health status, we used the 12-item general health questionnaire (GHQ-12) [46]. The validity and reliability of the Arabic version have been confirmed [47]. GHQ-12 scores were calculated using the GHQ scoring system, where: 0 = better than usual, 1 = same as usual, 2 = less than usual, and 3 = much less than usual. The cumulative score ranges from 0 to 36, with higher scores indicating higher degrees of disturbance of the general health status. Participants scoring 15 points or higher were considered to have a tendency toward psychological problems [48, 49].

### Statistical analysis

We used SPSS, version 21 to analyze the data. We set a 5% alpha level and 80% power in all of the statistical tests. We calculated the means and the standard deviations (SDs) for continuous variables, and percentages for categorical variables. To assess the categorical variables associated with depressive symptoms, we used the chi-squared test. We used the independent sample t-test to examine differences in the means of the continuous variables. We assessed the effects of the intervention on the outcome variables by calculating the percentage of mean change for each subject from pre-intervention to post-intervention then an independent t-test was employed to determine the difference in the mean of the percentage of the mean difference between the two groups. We did further analysis using two-way ANOVA analysis of covariance (ANCOVA) to compare the GHQ total score, depression and anxiety, social dysfunction, and loss of confidence between the intervention and the control group. All tests were two-tailed at a probability level of 0.05.

## Results

### Participants’ recruitment

**Fig. 1:**
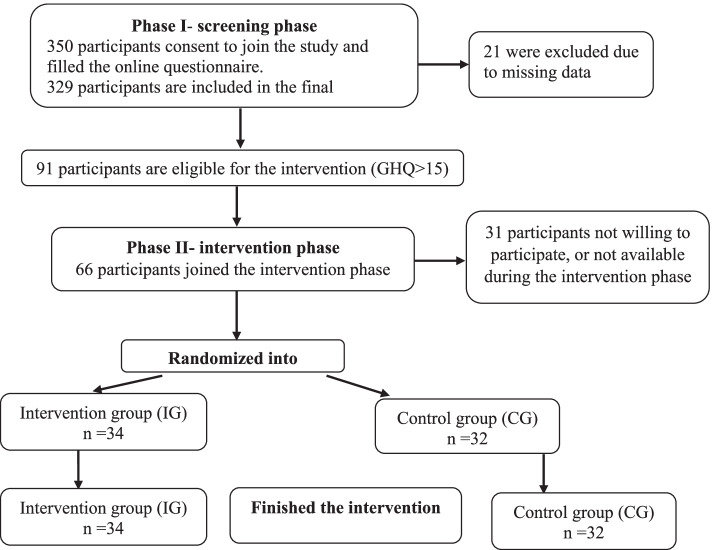
Shows the students’ recruitment flow chart

A total of 91 medical students from An-Najah National University, Palestine were included in the final analysis of the first phase of the study (screening phase). Twenty-one students were excluded mainly due to missing data. Then, a total of 66 students, out of 91 participants in the first phase of the study, satisfied the inclusion criteria of the intervention phase of the study and were randomly assigned to study groups after completion of baseline assessment. Thirty-one students were either not willing or not available to participate in the intervention phase as shown in Fig. 1.

### Students’ characteristics – screening phase

Table 2 shows the sociodemographic characteristics of our sample. The mean age of students was 19.5 ± 1.4 years, ranged from 17–27 years old. The analysis revealed that the vast majority of enrolled students (99.1%) were unmarried”. It was also revealed that nearly half of the students (50.5%) were living either in camps or in villages, and their family income was more than 5000 NIS per month by (44.3%). Furthermore, it was found that most of the students were not working (91.4%), and were living with their families by (91.4%).

**Table 2:** Students’ sociodemographic characteristics are presented in frequencies (n) and percentages (%)

Variables	Frequency (n)	Percentage %
College year		
First year	86	26.1
Second year	144	43.8
Third year	73	22.2
Fourth and fifth year	26	7.9
Living area		
City	164	49.2
Village + camps	165	50.8
Housing nature		
With family	293	90.2
Dorm	28	8.6
With relative	4	1.2
Personal status		
Single	326	99.1
Married	2	0.6
Other	1	0.3
Family income		
Less than 1000 NIS	19	5.8
1500–3000 NIS	124	38.2
3000–5000 NIS	38	11.7
More than 5000 NIS	144	44.3
Working status		
Regular job	3	0.9
Irregular job	25	7.6
Do not work	301	91.5
College fees		
Scholarship	20	6.1
Family	301	91.5
Other	8	2.4

### Medical history and life style of students

Most of the students (88%) stated that they are not smokers. Moreover, almost all the participated students (95.7%) were not suffering from chronic diseases and only (22.2%) had previous medical surgery. In regard to lifestyle variables; the results revealed the mean of sleeping hours is 7.5 ± 0.4 h/ day, screen time 6.5 ± 3.5 h/day, time spent in entertainment 4.6 ± 3.1 h/day. Furthermore, the analysis of the IPAQ survey showed that 62.1% of students were allocated in the low physical activity category, while 23.3% of students were classified as having a moderate physical activity level and only 14.5% of them have a high physical activity level.

### The mental health of students

**Fig. 2:**
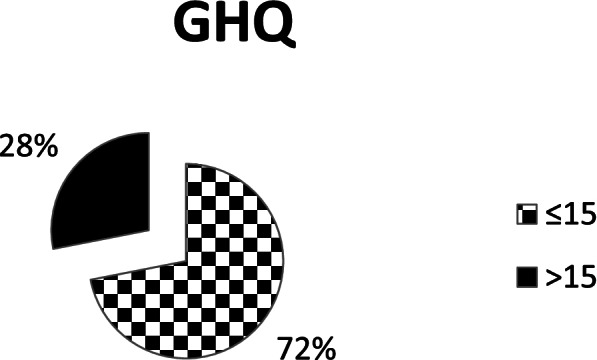
Students’ general mental health presented in percentages

The mental health of the participants is presented in Fig. 2. The mean of the GHQ score was (18.1 ± 7.7) ranging from 2- 36 points. For the GHQ subscales; the mean of depression and anxiety was 7.0 ± 2.8 ranging from 0–12 points, for social dysfunction the mean was 7.7 ± 3.4 ranged from 0–15 points, the mean for loss of confidence was 2.1 ± 2.0 ranged from 0–6 points.

### Nutritional status of students

Our data analysis showed that 9.3% of students were underweight, 65.5% had normal weight, and 18.6% were overweight, whilst only 6.6% of students were obese. In regards to adherence to the Mediterranean diet; the majority of students 63.3% had moderate adherence to MD, 24.5% had high adherence to MD, while only 11.2% of students had low adherence to MD.

### Risk factors associated with mental health

Our results revealed that neither good mental health nor poor mental health was associated with students’ sociodemographic characteristics, *p* > 0.05 using the chi-square test. As shown in Table 2, poor mental health was significantly associated with low physical activity, while there was no relationship between mental health with body mass index and adherence to MD score, *p* > 0.05 using independent t-test. In regard to lifestyle, there were significant relationships between good mental health status with a higher level of physical activity level, longer sleeping hours, and shorter entertainment time. Further analysis was done after categorizing students’ lifestyles into categories to determine the association with mental health showed a significant association between good mental health with being highly physically active, while the association was not significant with the adherence to MD, using the Chi-Square test, as shown in Table 3.

**Table 3:** The association between mental health with physical activity level and adherence to MD

	Mental health < 15	Mental health > 15	*P*-value
	n (%)	n (%)	
Adherence to MD			
High	32 (39.5)	49 (60.5)	0.657
Moderate	86(40.8)	125 (59.2)	
Low	13 (35.1)	24 (64.9)	
Physical activity (IPAQ)			
High Physical activity	25 (56.8)	19 (43.2)	0.011*^1^
Moderate physical activity	31 (43.7)	40 (56.3)	
Low physical activity	75 (35)	139 (65)	
	mean ± sd	mean ± sd	
Life style			
Sleeping hours (hours/ day)	7.7 ± 1.5	7.3 ± 1.5	0.039*^2^
Screen time study (hours/ day)	6.7 ± 3.7	7.3 ± 1.5	0.69
Entertainment time (hours/ day)	4.2 ± 2.6	5.0 ± 3.4	0.016*^2^

*sd* standard deviation

^*^^1^significant *p* < 0.05 using Chi-Square test. *^2^significant *p* < 0.05 using independent t-test

### Effect of treatment on GHQ total score, depression and anxiety, social dysfunction, and loss of confidence

**Fig. 3:**
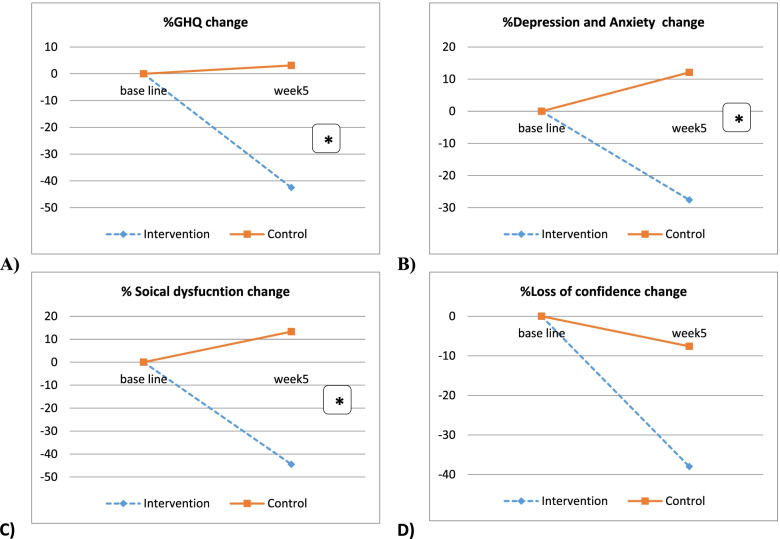
The difference of mean change between the control group and intervention group

A total of 66 students (15 males and 51 females) completely joined the intervention and were included in the final analysis. In general, the intervention had a significant effect on GHQ total score (*p* = 0001), depression and anxiety (*p* = 0.01), and social dysfunction (*p* = 0.000), but not on the loss of confidence (*p* = 0.13). Intervention and control groups were significantly different in mean scores of GHQ total, depression and anxiety, and social dysfunction at baseline, and 8-weeks follow-up (*p* < 0.05). However, intervention and control groups were not statistically significant in their loss of confidence scores at all points of measurement (baseline, and 8-weeks post-intervention) (Fig. 3) (Table 4).

**Table 4:** Effect of the intervention on Mental Health

Parameters	Intervention group (*n* = 34)	Control group (*n* = 32)	Group effect			Time effect			Interaction effect		
			**P**	***η***_***p***_^***2***^	**Power**	**p**	***η***_***p***_^***2***^	**Power**	**p**	***η***_***p***_^***2***^	**Power**
GHQ total Score											
Baseline	20.7 ± 6.4	22.5 ± 6.4	0.001	0.27	0.81	0.00	0.32	0.89	0.001	0.28	0.89
8-weeks	11.4 ± 6.7	22.1 ± 6.2									
Depression and Anxiety											
Baseline	7.8 ± 2.1	8.4 ± 2.3	0.01	0.28	0.87	0.001	0.11	0.78	0.001	0.225	0.89
8-weeks	5.1 ± 2.6	8.9 ± 2.5									
Social dysfunction											
Baseline	10.5 ± 3.4	11.0 ± 3.0	0.000	0.27	0.98	0.001	0.19	0.89	0.001	0.351	0.9
8-weeks	5.6 ± 4.1	12.0 ± 3.4									
Loss of confidence											
Baseline	2.4 ± 2.1	3.0 ± 2.1	0.13	0.05	0.34	0.17	0.04	0.364	0.13	0.035	0.327
8-weeks	1.4 ± 1.6	2.9 ± 2.1									

The findings also showed that there is a significant effect over time difference in mean scores of GHQ total score (*p* = 0.00), depression and anxiety (*p* = 0.001), and social dysfunction (*p* = 0.001). This indicates that responses for the two groups were statistically significant. However, the time effects for loss of confidence were not significant (*p* = 0.17) (see Table 4).

Testing for the interaction effect of time and group, the analysis showed that there is a significant group-by-time interaction found for GHQ total score (*p* = 0001), depression and anxiety (*p* = 0.001), and social dysfunction (*p* = 0.001). On the other hand, group-by-time interaction for loss of confidence was not statistically significant (*p* = 0.13) as shown in Table 4.

## Discussion

COVID-19 is a novel pandemic that has fatigued the lives of many people including students. Therefore, the novelty of the current study is that we targeted to estimate mental health problems among a sample of medical Palestinian students and investigate possible factors that might be related to developing mental health problems among them. We also aimed to examine the effectiveness of the cognitive-behavioral intervention on psychological problems among enrolled participants.

### The impact of COVID-19 on mental health and its associated factors

Our findings suggest a considerable negative effect of the COVID-19 pandemic on mental health, as 91 (28%) students obtained a GHQ-15 score > 15 points. This is in line with a recent study that found students experienced significant psychological distress [50]. Our findings are also consistent with a former study conducted by Islam et al. [51] who noticed unfavorable psychological responses (stress, anxiety, and depression) during the COVID-19 outbreak among Bangladesh undergraduates.

Various factors that are relevant to the undergraduates’ category may affect mental health including familial factors, debt, economic difficulties, poor quality of life, job losses and unemployment, and lifestyle factors [52]. In the current study, we observed that poor mental health status was significantly associated with low physical activity. This is in keeping with other studies, which demonstrate that physical activity and mental health are associated proportionally and it bears a positive effect on the education of youth [53].

Consistent with the findings of our study, several studies showed that mental health is associated with sleeping duration, such as the study by Chang et al., which showed that short sleep duration (< 7 h/ night) is positively associated with elevated depressive symptoms [54]. In contrast, two former studies found a U-shaped correlation between depressive symptoms and sleep duration [55, 56].

Though the mechanisms involved in the relationship between depressive symptoms and sleep duration are anonymous. First, people who sleep for a short period of time may have imperfect comfort and a higher perceived stress severity, which is considered a risk factor for depressive symptoms [57]. Second, former research found that university students who carry two alleles of low-expressing polymorphism of the serotonin transporter gene had more depressive symptoms in the existence of a permanent pattern of short night sleep [58].

Our findings showed that there was a significant inverse relationship between mental health and screen entertainment. These findings were in a parallel line with former studies conducted by Thomée et al. [59], and Chen [60], which showed that there is a correlation between addiction to mobile phones and mental health in dimensions of depression, psychosis, behavioral problems, and anxiety.

### The role of the CBT program on improving mental health symptoms among undergraduates

In terms of the second phase of the study, we found that the CBT program intervention had an effective effect on GHQ- total score, depression and anxiety, and social dysfunction among Palestinian medical students. The findings also demonstrated that there is a significant difference in mean scores between intervention and control groups in total GHQ, depression, anxiety, and social dysfunction, while no significant difference was found in the loss of confidence. This points out that although students in the intervention group scored lower in loss of confidence, they were not different and did not do better than their counterparts in the control group.

Nevertheless, the findings are consistent with the literature that supports the efficacy of CBT in treating depression and anxiety in Parkinson’s disease patients [61]. Moreover, the current study builds on the preliminary findings of Hamdan and colleagues [41] who explored that using CBT program lessened the depressive symptoms among Jordanian university students. The results of this investigation are also in accordance with a former study [62] which found that using CBT programs reduced the depressive symptoms among adolescents, and CBT had a higher impact on depression in comparison to other types of intervention.

Our findings should be interpreted with caution given the limitations of our study. Firstly, the study only included participants from one university which means that these results don’t exemplify all medical students in Palestine. Secondly, online assessments inherently carry bias and are less reliable. Nonetheless, our study is the first of its kind to estimate mental health problems and their associated factors among Palestinian medical students, moreover, it can be considered the first study in Palestine that discuss the efficacy of using cognitive-behavioral therapy program with university students having mental health problems. Future studies should be conducted on a larger and more representative sample for a better understanding of the effectiveness of the program.

## Conclusion

The present study evaluated and measured the mental health of Palestinian medical students during the online learning period using the GHQ-12 questionnaire. We found that the prevalence of poor mental health status among our sample was 28%. Further analysis revealed that students’ mental health status is significantly associated with physical activity level, duration of sleeping, and entertainment time. The study also provides research-based evidence of the effectiveness of using cognitive-behavioral therapy with university students in Palestine who have mental health problems. The study revealed a decrease in the level of total GHQ score, depression, anxiety, and social dysfunction. Future studies should estimate the sustainability of the impact of this intervention over time and determine if students need continuous support.

## Supplementary Information

**Additional file 1.** 

## Data Availability

Data is available when requested from the corresponding author.
